# Functional Superior Mesenteric Artery Syndrome Induced by an Optional IVC Filter

**DOI:** 10.1155/2019/6543934

**Published:** 2019-08-14

**Authors:** Adam Shen, Dina Tabello, Nishant Merchant, Joseph V. Portereiko, Alfred Croteau, Jonathan D. Gates

**Affiliations:** University of Connecticut, Hartford Hospital, 80 Seymour Street, Hartford, CT 06102, USA

## Abstract

This patient suffered multiple injuries in a motor vehicle crash. She had an optional IVC filter placed in the usual fashion and location which resulted in a functional obstruction of the third part of the duodenum much as one would expect with a Superior Mesenteric Artery (SMA) syndrome. The symptoms persisted over the sixteen-day filter dwell time and resolved completely with the retrieval of the filter.

## 1. Introduction

This is a presentation of a victim of a high-speed motor vehicle crash who underwent multiple operative interventions for both intra-abdominal injury and fractures in the upper and lower extremities. An inferior vena caval (IVC) filter was placed for prophylaxis against pulmonary embolism as her risk was determined to be high and enoxaparin would be held for a brief period of time due to a combination of an intracranial bleed and frequent trips to the operating room. She had return of bowel function following a small and large bowel resection only to develop an SMA-like syndrome with duodenal obstruction. Cross-sectional imaging and an upper GI series documented the duodenal obstruction through the compression of the duodenum between the IVC filter and the SMA. This obstructive pattern resolved with the removal of the IVC filter.

## 2. Case Presentation

The patient was a 23-year-old woman who was otherwise healthy when she was involved in a high-speed motor vehicle crash. Two of the passengers in the other vehicle died at the scene. The patient was intubated at the referral hospital for a persistently low Glasgow Coma Score (GCS) of 8. On subsequent radiographic evaluation, she was noted to have a small subdural hematoma, a moderate amount of hemoperitoneum thought to be related to a possible splenic laceration, an L5 end-plate fracture, a right humeral fracture, a right femur fracture, and an open right patellar fracture. During the transfer via helicopter to a higher level of care, she became hypotensive and was noted to be a transient responder to blood products dictating the need for an emergent trip to the operating room upon arrival at the tertiary care center.

An exploratory laparotomy revealed a small bowel mesenteric injury near the terminal ileum, and 30 cm of small bowel was resected and left in discontinuity. A rent in the sigmoid colon was noted, and a 10 cm segment was resected and also left in discontinuity. There was a minor Grade I injury of the spleen. At no point was the duodenum manipulated or a Kocher maneuver performed. The abdomen was left open with an ABThera device in place, an external fixator was placed on the right femur fracture, and a closed reduction and splinting was performed for the right humeral fracture.

She was returned to the operating room on POD#2 to reestablish bowel continuity with a small bowel and then sigmoid colon reanastomosis followed by abdominal closure. She was found to have an end-plate fracture of L5; however, this was determined to be stable, hence there was no need for a brace for immobilization. The external fixator was removed and the femur fracture definitively repaired at the time of final laparotomy. The right humeral fracture was internally fixated on POD#10/12.

The inferior vena caval filter (DENALI Vena Cava Filter, Bard Peripheral Vascular Inc., Tempe, AZ) was placed on HD#5 as a prophylactic intervention in an individual who would be at high risk for pulmonary embolism given her extensive extremity injuries and multiple trips to the operating room with a known intracranial bleed from trauma. The intermittent use of subcutaneous enoxaparin would place the patient at significant risk of pulmonary embolism.

**Figure 1:**
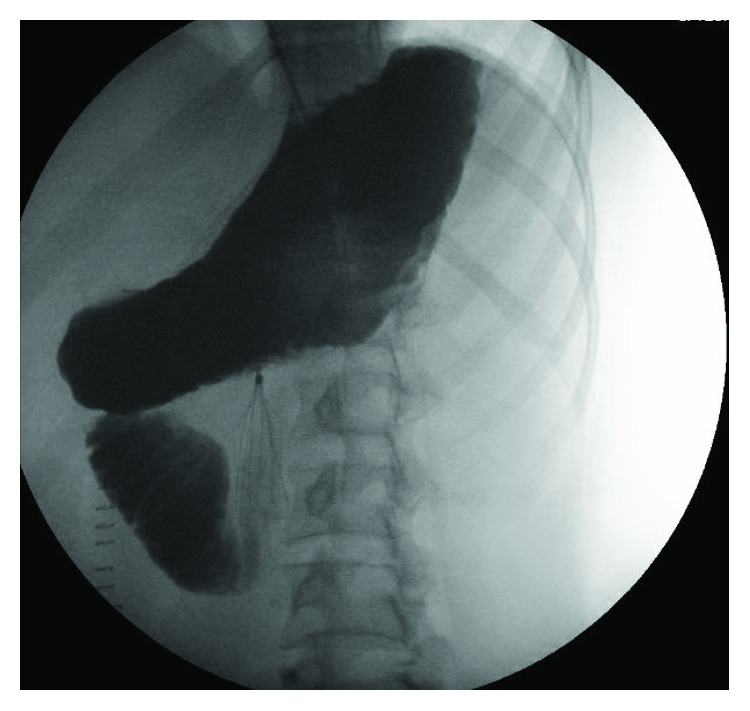
The upper gastrointestinal series demonstrates a high-grade obstruction of the third part of the duodenum across from the IVC filter.

**Figure 2:**
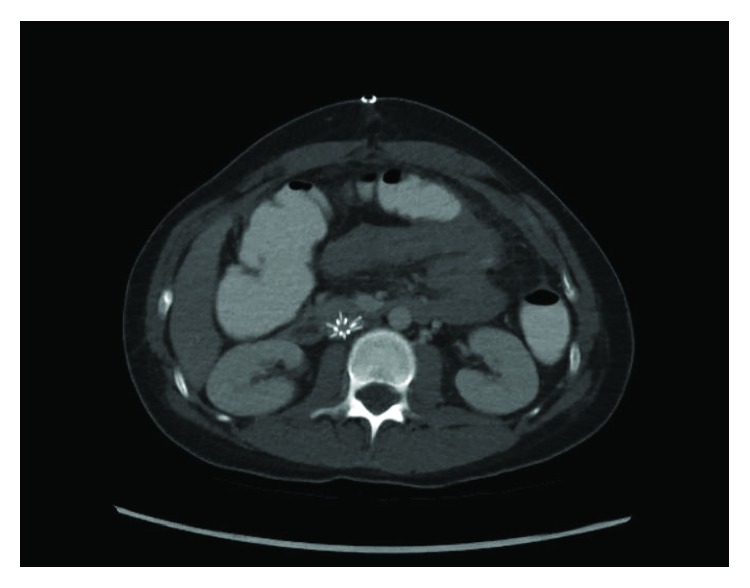
This figure demonstrates the IVC filter posteriorly and the SMA and SMV anteriorly with the third part of the duodenum sandwiched in between.

**Figure 3:**
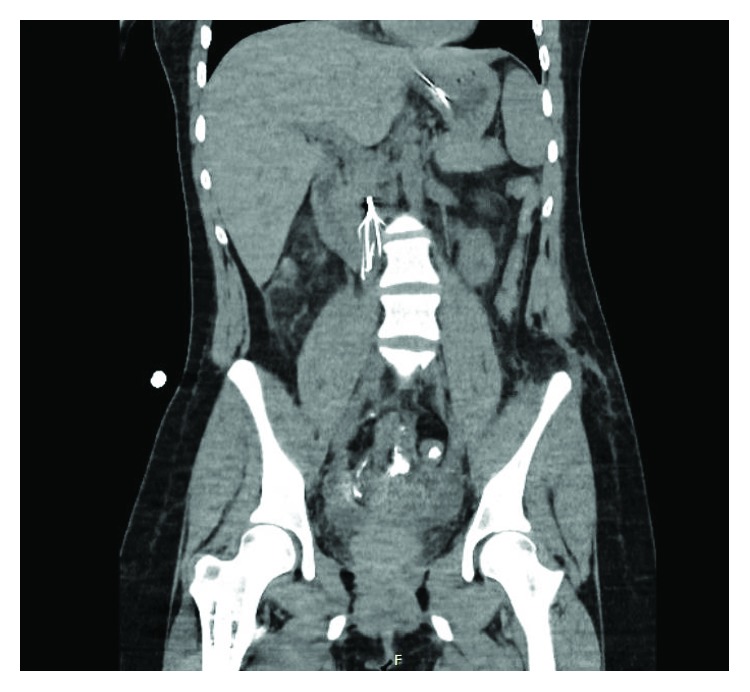
This figure demonstrates the relationship between the IVC filter and the subsequent dilatation of the more proximal second part of the duodenum as a result of the obstruction. Note a small glimpse of the nasogastric tube in the stomach for decompression and the absence of a duodenal hematoma.

**Figure 4:**
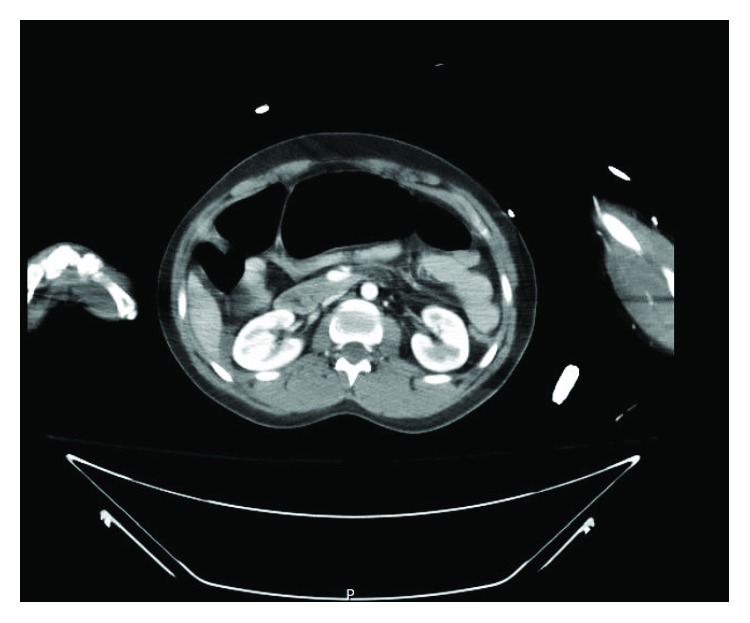
This is the CT scan obtained on the day of admission following the motor vehicle crash. Note the absence of any radiographic suggestion of duodenal obstruction by the SMA. Also note the relatively small size of the IVC as a reflection of volume depletion on presentation and the free blood in the right gutter.

Her oral intake was sparse and poorly tolerated with intermittent nausea and vomiting until she demonstrated the need for nasogastric decompression due to voluminous vomiting on POD#12/14. A KUB at that time was unrevealing and demonstrated minimal gas, if any, in the bowel. On POD#16/18 and with an IVC filter dwell time of 16 days, an upper GI study demonstrated an almost complete cut-off of the contrast flow beyond the second to third part of the duodenum (Figure 1). The obstruction to the flow of oral contrast correlated with the location of the IVC filter in the infrarenal IVC to create a functional obstruction within the duodenum similar to an SMA syndrome. Computerized tomographic scan cross-sectional imaging shows a pinching mechanism between the right-sided IVC filter and the left-sided SMA with the third portion of the duodenum compressed between the two structures (Figures 2 and 3). This corresponds to the area of complete occlusion of the third part of the duodenum. A review of the abdominal-pelvic CT scan on the day of admission (Figure 4) failed to show any preexisting evidence for SMA syndrome, and it was ascertained that there was no history of oral intake intolerance prior to the injury. There is some free blood in the abdomen on that original CT scan but no evidence for duodenal hematoma. The IVC filter was removed after a dwell time of 18 days, and clinically, the patient continued to put out a large amount of fluid from the nasogastric tube.

**Figure 5:**
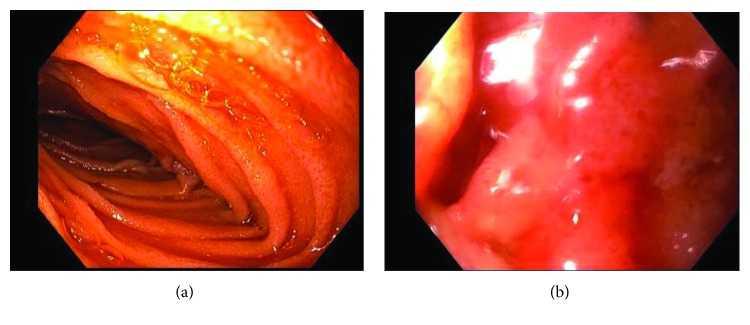
(a) This is the view on the upper endoscopy of the distal second part of the duodenum with partial obstruction. (b) This is the same upper endoscopy performed as in (a) at the level of the third part of the duodenum with edema and obstruction to the point where the endoscope could not be advanced beyond the obstruction.

**Figure 6:**
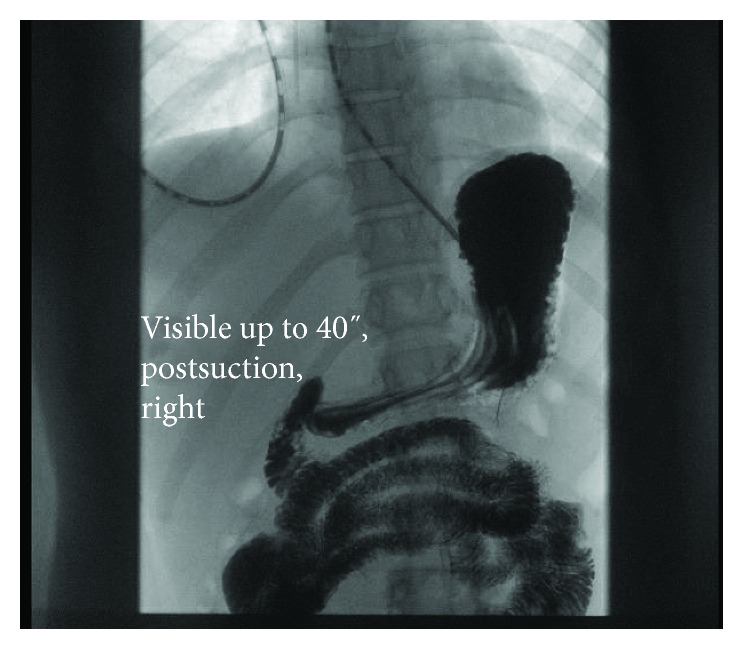
The IVC filter has been removed and the upper gastrointestinal series some 10 days later demonstrates complete resolution of the duodenal obstruction.

An upper endoscopy was performed five days after the IVC filter had been removed, yet the duodenal edema and obstruction persisted (Figures 5(a) and 5(b)) requiring NG tube decompression for another four days. At that point, an upper GI series was repeated (Figure 6) and that demonstrated complete resolution of the previously documented duodenal obstruction. The nasogastric tube was removed, and the patient resumed a diet without further issue.

## 3. Discussion

Common complications from optional IVC filters have included filter misplacement, filter migration, filter strut perforation of the IVC, strut penetration of the duodenum and aorta, and strut fractures [1–7]. This is an unusual case of duodenal obstruction as a result of the presumed inflammation and edema from an IVC filter in the infrarenal vena cava creating a pinching mechanism between the IVC filter and the SMA with resultant obstruction of the intervening third part of the duodenum. The duodenal obstruction persisted for an additional ten days after the IVC filter was removed, presumably as the edema that was identified on the upper endoscopy resolved over time. The IVC filter limbs impact on the wall of the inferior vena cava and create an inflammatory reaction in the surrounding perivascular tissues. The duodenum was presumably incorporated into that inflammation, and this presented as mucosal edema. Once the source of the inflammation was removed, the edema resolved in due time.

The more common cause of a similar duodenal obstruction is from the classic Superior Mesenteric Artery (SMA) syndrome. The normal angle of the SMA off the aorta ranges from 38 to 65 degrees with a cushion from the intervening mesenteric fat [8]. The higher the body mass index (BMI), the greater this angle will become. The distance from the anterior aorta to the undersurface of the SMA is known as the aortomesenteric distance, and this usually ranges from 10 to 28 mm Hg [9]. In this young woman, the BMI was approximately 25 with an SMA angle of 23 degrees and an aortomesenteric distance of 6 mm. The BMI category is on the lower end of mild obesity, and the angle and aortomesenteric distance are also on the low end and would in some cases be consistent with that of SMA syndrome. She never had any symptoms of the SMA syndrome preinjury and continues postinjury to be free of symptoms in clinic follow-up. In fact, the measurements of the SMA angle and the distance of the SMA to the anterior border of the infrarenal aorta remained the same from the initial CT scan of the abdomen on admission to subsequent CT scans of the abdomen performed to investigate the cause of her inability to tolerate any oral intake during her hospitalization.

This case represents a reversible complication of an IVC filter that mimics the clinical presentation of a functional SMA syndrome with an alternative treatment pathway. The IVC filter in and of itself would be insufficient to create the functional appearance of an SMA syndrome because the IVC filter is posterior to the duodenum and pushes it forward without a fixed and rigid structure placed anteriorly to complete the compression. In this case, the normally positioned but more left-sided SMA provided that anterior fixed structure that completed the static anterior posterior compression. A review of the cross-sectional imaging demonstrates the sequential compression of the intervening duodenum by the anterior displacement of the duodenum by the IVC filter, the posterior compression by the bundle of the SMV and SMA, and the anterior displacement of the duodenum by the aorta. Because of the iatrogenic placement of the IVC filter, the third part of the duodenum became obstructed in what should be considered a variant from the classic SMA syndrome in which the unyielding structure was not the aorta alone but the combination of the IVC filter in the inferior vena cava and the SMA. The IVC filter was positioned appropriately in the infrarenal IVC at the level of the second and third lumbar vertebrae.

Certainly, the SMA syndrome has been described from a number of acute causes that include significant weight loss, debilitating illness, and trauma, as in this case [10, 11]. The common factor in all of these cases is weight loss with a reduction in the size of the cushioning of the mesenteric fat pad between the aorta and the SMA. She was initiated early on in her course with a regimen of total parenteral nutrition when it was apparent that her bowel obstruction was not opening up. There was little expectation that the ten day period of TPN would be adequate to restore the quantity of the mesenteric fat pad sufficiently to cure the SMA syndrome. However, the treatment here was not just further nutrition but was removal of the IVC filter.

There are reports of patients who have had mild degrees of the SMA syndrome correlated with position with relief obtained with lying prone, knee-chest, or left lateral decubitus position, all of which open up that crucial aortomesenteric window [12]. She was not confined to bed rest, but her constellation of injuries including her femur fracture limited her mobility to some degree and she was often found sitting up in bed on rounds.

There have been prior reports of the SMA syndrome from unusual intrinsic causes as in a juxtarenal aneurysm with anterior enlargement and entrapment of the third part of the duodenum between the neck of the aneurysm and the overlying SMA [13]. It is important to recognize these variations in the SMA syndrome as part of the differential in bowel obstruction and not assume that the lack of return of bowel function is then ascribed to postoperative ileus or constipation. Fortunately, this IVC filter was an optional filter, as the majority of them are nowadays; otherwise, it would have required an open procedure for removal and resolution of the obstruction.

The fact that this is not a more commonly reported finding with the use of IVC filters must represent the perfect storm of preexisting anatomy, body habitus, relative immobility from injury, the variable location of the relationship of the duodenum to the IVC, and the location of the tines of the IVC filter.
